# Dental caries experience and treatment needs of an adult female population in Nigeria

**DOI:** 10.4314/ahs.v17i3.34

**Published:** 2017-09

**Authors:** Folake Lawal, Omolola Alade

**Affiliations:** 1 Department of Periodontology and Community Dentistry, University of Ibadan and Department of Periodontology and Community Dentistry, University College Hospital, Ibadan; 2 Department of Periodontology and Community Dentistry, University College Hospital, Ibadan

**Keywords:** Adults, dental caries, DMFT, female, prevalence, treatment needs

## Abstract

**Background:**

Experience and awareness of adult females concerning dental caries is important in its prevention particularly in children because of their natural role as care givers.

**Objectives:**

To determine the prevalence of dental caries and treatment needs in an adult female Nigerian population.

**Methods:**

In this cross-sectional study, adult females attending outreach programmes were examined for dental caries using the Decayed Missing and Filled Teeth caries index (DMFT). Socio-demographic variables were also recorded and statistical analysis done with SPSS software.

**Results:**

A total of 430 females aged 16 to 59 years participated in the outreach programme out of which 109 (25.3%) had a DMFT score > 0. Mean DMFT was 0.7 ± 1.6. Fifty-five (12.8%) participants had decayed teeth, 78 (18.1 %) had missing teeth and 10(2.3%) had filled teeth. The treatment need was 34.3%, restorative index was 13.3% and significant caries index was 2.0. There were significant differences in caries experience based on age, marital status and educational qualifications of participants p < 0.05.

**Conclusion:**

The prevalence of dental caries among the study group was low but the treatment need was high. Younger females, singles and those with lower educational qualifications had a higher dental caries experience.

## Introduction

Dental caries is a multifactorial biofilm disease^1^ that most commonly has its onset in childhood. The prevalence of dental caries has been documented as 13.1% to 36.3% in children in Nigeria and other parts of the world^2^–^4^. Higher prevalence of 40.0% to 68.5% had been reported in Brazil^5^, Asia^6^ and Finland for adults^7^, thus suggesting that there is no age at which an individual becomes immune to dental caries^8^. Global data showed that dental caries still remains a problem of public health significance^9^ as increased life expectancy and longer retention of teeth have translated into an increase in the burden of dental caries in adulthood^10^. The burden of dental caries has equally been associated with the caries experience of mothers; the Decayed Missing Filled Surfaces (DMFS) score of mothers was reported to have a significant positive correlation with the caries status of their children^11^. Similarly, the vertical transmission of *Streptococcus mutans* caries causing bacteria from care givers to children has been documented^12^. Furthermore, women are also more likely to experience dental caries than men^13^–^15^. This places women at a central position and as targets for oral health promotion; however, holistic approach at preventing dental caries at the community level focused at adult females who may also double as mothers and key care-givers in the society has not been fully explored. This becomes more important in our environment and in other developing countries where many children live with unmet dental needs resulting from dental caries^2^ more so that dental caries significantly impact negatively on the quality of life^16^. For planning to be adequately achieved, there is a need to provide baseline data about this highly preventable oral disease in the female adult population in order to set aright appropriate strategies required for its prevention. The aim of this study therefore was to evaluate the dental caries experience and its treatment needs among adult females at a community level in Nigeria and provide the required exploratory data for subsequent primary prevention.

## Methods

This descriptive cross-sectional study was conducted in the metropolis of Ibadan. Ibadan is the largest city in West Africa and the capital city of Oyo State in South-West Nigeria. The study population consisted of adult females who participated in dental outreach programmes conducted by dental public health professionals from one of the largest university teaching hospitals in the country. The study participants were recruited from dental outreach programmes targeted at promoting oral health among the populace as a means of primary prevention over a period of six weeks. Furthermore, they simulated the type of population to whom interventions will be directed at, thus an appropriate study population.

After educating the study participants about their oral health, the purpose of the study was explained and only the females present and who consented to participate were recruited for the study. The outcome of interest was caries experience as determined by DMFT > 0, decayed tooth, missing tooth due to caries, filled tooth and tooth tender to percussion. The exposures were age, marital status and educational qualification.

The bio-data of the study participants was obtained before oral examination was conducted. The occupation of the respondents was grouped into occupational classes using a modification of OPCS, which was modified for this environment in a previous study^17^. The occupational classes consisted of skilled workers, unskilled workers and dependants. The educational level was recorded as secondary school or less, post-secondary (those in colleges and other schools higher than secondary but not university) and tertiary (university). Intra-oral examination was conducted according to the standards of the World Health Organization^18^ and the caries experience of this population was assessed using DMFT index. Information on the Decayed (D), Missing (M) and Filled (F) teeth due to dental caries was charted on the oral examination assessment form. Presence or absence of pain from the teeth due to dental caries was also charted as present or absent. Oral examination was conducted by two trained and calibrated examiners prior to the study and inter and intra-examiner variability was determined at this period and during the course of the study. The inter examiners variability ranged from 0.74 to 0.79. The D represented the decayed teeth M; missing teeth due to caries, F; filled teeth due to caries and the DMFT score was the addition of the D, M and F for an individual. The total DMFT score for the population was the addition of all individual DMFT scores. The mean DMFT was the total DMFT score divided by the total number of persons examined. The treatment need of the population denoted the percentage of those with caries experience that required treatment and it was calculated as D/DMFT * 100. The Met Need Index (MNI), an indication of treatment received by an individual, was obtained using the formula: MNI = M+F/DMFT. Restorative Index RI, a reflection of the restorative care of those who had suffered from dental caries, was calculated using: RI = F/D+F * 100. The significant caries index (SiC) was obtained as the mean DMFT of one third of the participants with the highest DMFT score. Presence or absence of tooth/teeth tender to percussion was also recorded.

Ethical approval for the study was obtained from the University of Ibadan and University College Hospital, Ibadan Institution Review Board.

Data obtained was analyzed using SPSS version 21. Oral examination findings were re-categorized as presence or absence of teeth tender to percussion, decayed, missing or filled teeth. Chi square statistics was used to determine associations between variables and the cut off level of statistical significance set at 5%.

## Results

### Socio-demographic characteristics of respondents

The participating females were 430 in number. The age range of the participants was 16 to 59 years, the median age was 49 years while the mean age was 45.1 ± 10.3 years. The majority of the study participants (94.7%) was married and 87.4% had post-secondary or higher education (Table 1).

**Table 1:** Socio-demographic characteristics of respondents

Variable	Frequency	%	SiC
**Age years**			
< 35	58	13.5	2.4
35–44	84	19.5	2.1
< 45	288	67.0	1.8
**Marital status**			
Single	8	1.9	3.7
Married	407	94.7	1.9
Widowed	15	3.5	1.6
**Educational qualification**			
Secondary or less	54	12.6	2.8
Post-secondary	289	67.2	1.5
Tertiary	87	20.2	3.0

SiC - Significant Caries Index

### Dental caries experience

About a quarter, 109 (25.3%), had DMFT > 0 and mean DMFT score was 0.67 ± 1.6. The mean decayed teeth DT was 1.8 ± 1.1, missing teeth MT was 2.2 ± 2.0 and filled teeth FT was 1.5 ± 1.0.

### Decayed, missing and filled teeth

The number of decayed teeth per individual ranged from one to five and a tooth or more was decayed due to caries in 55 (12.8%) respondents. The total number of decayed teeth was 98, thus making 34.3% of total DMFT. The number of missing teeth due to caries per individual ranged from 1 to 14 and the total number of missing teeth was 173 making 60.5% of total DMFT. A missing tooth was found in 78 (18.1%) respondents. Only 10 (2.3%) respondents had filled tooth/teeth due to caries and the number of filled teeth ranged from one to four per individual. The total number of filled teeth was 15 constituting 5.2% of total DMFT.

### Treatment need

The treatment need was 34.3%, while the Met Need Index was 0.66 and the restorative index was 13.3%. The significant caries index was 2.0.

### Teeth tender to percussion

Less than 10% (33) had at least a tooth tender to percussion with 20 (60.6%) having a tooth tender, 7 (21.2%) had two teeth, 4 (12.2%) females had three teeth, 1 (3.0%) had four teeth tender to percussion and more than 4 teeth were tender to percussion in 1 (3.0%) person.

### Socio-demographic characteristics of respondents and caries experience

#### Age

There was a significant age difference between those who had DMFT of 0 (p = 0.048) and those whose DMFT was > 0 (p < 0.001). Most of the participants with DMFT > 0 were 44 years old or less. There was no significant association between age and having a missing tooth, and filled tooth (Table 2.)

**Table 2:** Socio-demographic characteristics and caries experience of respondents

Sociodemographic	Age N (%)			Total	χ^2^	p value
variable/DMFT index	< 35years	35 –44 years	≥ 45	years	N (%)	
DMFT = 0	36 (62.1)	62 (73.8)	223 (77.4)	321 (74.7)	6.059	0.048*
DMFT > 0	22 (37.9)	22 (26.2)	65 (22.6)	109 (25.3)		
D = 0	38 (65.5)	74 (88.1)	263 (91.3)	375 (87.2)	28.887	< 0.001*
D > 0	20 (34.5)	10 (11.9)	25 (8.7)	55 (12.8)		
M = 0	52 (89.7)	66 (78.6)	234 (81.2)	352 (81.9)	3.057	0.217
M > 0	6 (10.3)	18 (21.4)	4 (18.8)	78 (18.1)		
F = 0	56 (96.6)	82 (97.6)	282 (97.9)	420 (97.7)	0.397	0.820
F > 0	2 (3.4)	2 (2.4)	6 (2.1)	10 (2.3)		
**Marital status/DMFT index**	**Single**	**Married**	**Widowed N (%)**	**Total**	**χ^2^**	**p value**
	**N (%)**	**N (%)**				
DMFT = 0	3 (37.5)	309 (75.9)	9(60.0)	321 (74.7)	7.884	0.019*
DMFT > 0	5 (62.5)	98 (24.1)	6 (40.0)	109 (25.3)		
D = 0	5 (62.5)	357 (87.7)	13 (86.7)	375 (87.2)	4.476	0.107
D > 0	3 (37.5)	50 (12.3)	2 (13.3)	55 (12.8)		
M = 0	4 (50.0)	338 (83.0)	10 (66.7)	352 (81.9)	8.186	0.017*
M > 0	4 (50.0)	69 (17.0)	5 (33.3)	78 (18.1)		
F = 0	8 (100.0)	397 (97.5)	15(3.6)	420 (97.7)	0.579	0.7498
F > 0	0 (0.0)	10 (2.5)	0 (0.0)	10 (2.3)		
**Educational Qualification/DMFT**	**Secondary**	**Post secondary**	**Tertiary**	**Total**	**χ^2^**	**p value**
**index**	**/less**	**N (%)**				
	**N (%)**		**N (%)**			
DMFT = 0	32 (59.3)	229 (79.2)	60 (69.0)	321 (74.7)	11.461	0.003*
DMFT > 0	22 (40.7)	60(20.8)	27 (31.0)	109 (25.3)		
D = 0	33 (61.1)	226 (92.0)	76 (87.4)	375 (87.2)	39.024	< 0.001*
D > 0	21 (38.9)	23 (8.0)	11 (12.6)	55 (12.8)		
M = 0	48 (88.9)	241 (83.4)	63 (72.4)	352 (81.9)	7.481	0.024*
M > 0	6 (11.1)	48 (16.6)	24 (27.6)	78 (18.1)		
F = 0	52 (96.3)	282 (97.6)	86(3.6)	420 (98.9)	0.993	0.609
F > 0	2 (3.7)	7 (2.4)	1 (0.0)	10 (1.1)		

* statistically significant

#### Marital status

Participants who were single had more missing teeth due to caries than others (p = 0.017) and higher dental caries experience than others (p = 0.019). No significant association was found between marital status and having a filled tooth (Table 2).

#### Educational qualification

Women with secondary education had higher caries experience (p = 0.003) and more decayed teeth (p < 0.001) than those with university or post-secondary education. Respondents with university education had more missing teeth due to dental caries than the others (p = 0.024). There was no significant association between educational qualification and having filled tooth (Table 2).

## Discussion

This study sought to describe the dental caries experience and treatment needs of an adult female population in Nigeria in a bid to provide baseline information and a template on which the promotion of oral health as regards dental caries among women in this environment will be based. We found that 25.3% of women in this study had experienced dental caries. This is much less than the prevalence of dental caries experience reported among female populations in India^19^, Korea^20^ and Spain^21^. The proportions of women with dental caries experience in those studies were 63.3%, 91.6% and 93.2% respectively. However, our finding supports previous caries epidemiology studies in Nigeria that show that the prevalence of dental caries in Nigeria remains relatively low^22^.

The mean DMFT score among the women in this study was 0.67. Studies in other countries had reported mean DMFT scores ranging from 3.09 to 7.89 among women of similar age to our study population^19^,^21^,^23^. Furthermore, Omitola and Arigbede^24^ also reported a mean DMFT of 3.88 among Nigerian women in a hospital based study. This is not surprising as it is expected that mean DMFT among patients presenting at a dental centre would be higher than that obtained from a community based study like ours. However, the significant caries index was suggestive of a relative severity among those affected despite the low prevalence.

Missing tooth constituted the major component of the DMFT index, which is a reflection of fatality resulting from tooth extraction due to dental caries. A high proportion of teeth missing due to caries was also reported in a Hungarian national study of 2,923 females of all ages^25^. However contrary findings had been reported in India^19^,^23^ Spain^21^ and Thailand^26^ where the greatest burden of dental caries experience among women was due to decayed teeth. The differences between those studies may be attributable to the makeup of the study population as well as the level of awareness of the participants. Our findings suggest that women do not present early enough for dental care, evident by 33 (7.7%) of the women in this study who were in pain due to caries — they had at least one tooth that was tender to percussion. This also suggests that despite the relatively low prevalence of dental caries, carious lesions remain untreated until they become symptomatic and require extractions. This oral health behaviour, is similar to what had been reported in children and adults in Nigeria^27^,^28^. This was also corroborated by the very low restorative index in the present study. There is, thus, a dire need to educate mothers on the importance of taking up preventive dental care and routine dental checkup, which are also essential in the prevention of dental caries in this part of the world. This would likely help to improve the oral health related quality of life of both women and children.

A significant association was found between age and caries experience with the younger women experiencing more dental caries than the older ones. This was similarly observed by Lee et al.,^20^ where caries experience increased with age; 84.6% in the 19 to 34 years, 85.8% in the 35 to 44 years, 90.3% in the 45 to 54 years and 93.5% among the 55 to 64 years age groups in their study. Also, observed in the present study was that younger females had a higher proportion of untreated decayed tooth compared to older women. Kumar et al.,^23^ equally reported that mean DMFT increased with age of women in their study.

Marital status was significantly associated with dental caries in this study; a higher proportion of single women had experienced dental caries and had more missing teeth due to caries when compared to married and widowed females, this may be partly attributable to their lifestyles and is probably a factor of age. Those with lower educational qualifications had a higher proportion of decayed teeth and dental caries experience compared to those with higher educational qualifications. This was similarly reported by Rakchanok et al.,^26^ in a study in which women with high school education had a 2.8 (1.2 to 6.3) fold odd of having dental caries over those with college education when pregnancy status, age group, occupation and health insurance status were accounted for. However, a higher proportion of females with tertiary education had more missing teeth than others. Finding that those with higher educational qualifications had less decayed teeth, dental caries experience and more missing teeth is a reflection of impact of education on awareness level about oral health. This probably made them seek dental treatment compared to those with lower educational qualifications who did not thereby having higher proportion of untreated decayed teeth. However, their late presentation for dental care left them with tooth extraction as the only feasible treatment option. This is a subject of concern and a need to intervene to improve the dental care seeking behavior of females irrespective of educational status as early presentation and preventive dental care still remains the key to reducing fatalities in those already affected by dental caries.

## Limitations

A limitation of this study was the fact that it was based on participants at outreach dental services and thus may not be representative of females in every Nigerian community. However as observed by Kassim et al.,^29^ outreach programmes are often the only means of obtaining data in hard to reach communities in resource poor countries.

## Conclusion

The prevalence of dental caries among the study group was low but the treatment need was high. Younger females, singles and those with lower educational qualifications had a higher dental caries experience.
